# Psychological and behavioral characterization of suicide ideators and suicide attempters in adolescence

**DOI:** 10.3389/fpsyt.2022.1009460

**Published:** 2022-10-10

**Authors:** Chiara Coci, Roberta Invernizzi, Luca Capone, Erica Casini, Marika Orlandi, Paola Galli, Ilaria Rossi, Ottaviano Martinelli, Renato Borgatti, Martina Maria Mensi, the Northern Italian Suicidality Research Group

**Affiliations:** ^1^Department of Brain and Behavioral Sciences, University of Pavia, Pavia, Italy; ^2^Child Neurology and Psychiatry Unit, ASST Lecco, Lecco, Italy; ^3^Department of Medicine and Surgery, University of Milano-Bicocca, Milan, Italy; ^4^Child Neurology and Psychiatry Unit, IRCCS Mondino Foundation, Pavia, Italy

**Keywords:** adolescence, suicide attempt (SA), attraction to life, repulsion by life, suicidal ideation, assessment

## Abstract

Suicide is a global cause of death, a chronic disability, and a significant public health problem. Recent works emphasize the importance of differentiating people with suicide ideation (SI) and people with suicidal attempts (SA), so we conducted a clinical cross-sectional study to better characterize the features most associated with SA. We enrolled 88 adolescents (77 females) from 12 to 18 years of age (*M* = 15.21, *SD* = 1.63) admitted to Northern Italian Child Neurology and Psychiatry Service who presented SI and/or SA. We conducted an assessment using the Columbia-Suicide Severity Rating Scale, and divided participants into two groups: adolescents with SA, and adolescents with thoughts about killing themselves which may include a plan but no suicidal attempts (SI). We found that the SA group showed greater severity of SI [*t*_(86)_ = −3.485, *p* < 0.001], higher levels of subjective depression [*t*_(70)_ = −2.65, *p* = 0.01)], and a higher prevalence of personality disorders [$\chi_{\left(3\right)}^{2}$ = 8.775, *p* = 0.032] than the SI group. Both groups presented a prevalence of internalizing problems compared to the externalizing ones in the Youth Self-Report (YSR). Higher scores on YSR internalizing problems correlate positively with the “Repulsion by Life” subscale of the Multi-Attitude Suicide Tendency (MAST) Scale in both SA and SI groups (*p* = 0.41 and *p* = 0.67, respectively), while low levels of the MAST “Attraction to Life” subscale appear more often in the SA one (*p* = −0.71). In conclusion, results showed that some features (e.g., prevalence of personality disorders, SI intensity, and subjective depression) might help clinicians distinguish between patients with SI and those with SA and support the importance of carefully pursuing this distinction in research.

## Introduction

Suicide is recognized as an important cause of death and chronic disability worldwide. In 2019 it was the fourth leading global cause of death in the population aged 15–29 years and, during the COVID pandemic, there was an increase in suicidal ideation (SI) internationally (1). Both successful and failed suicide attempts (SA) have a major impact on public health by also afflicting families and communities (2, 3). Despite numerous investments in the prevention of such behaviors, in Italy in 2021 the World Health Organization reports an increase in suicide rates (4), and many challenges to research and prevention remain open in this area. In Europe, UNICEF estimated that suicide is the second cause of death in adolescence (5) and in Italy the suicide rate in 2021 was 1.71 per 100,000 person-years among males and 0.65 among females (6).

Several authors pointed out some gaps in the literature related to the characterization of individuals at risk for SA. Most studies are focused on the suicidal subject neglecting the differentiation between people with SI and those with SA and the factors that promote the transition from one condition to the other (7, 8). It is well known that most individuals who experience SI do not perform an actual act (9, 10), and several studies hypothesize that SI and SA correlate with different predictors and also differ from those of actual suicide deaths (11, 12). Finally, most studies differentiate samples into “suicidal” and “non-suicidal” groups, facilitating confusion between characteristics related purely to SI (which every attempter experiences) and factors specifically related to SA (13).

Therefore, our study aims to explore the features of Italian adolescent patients who present SI and SA through scales more sensitive to detect differences between SI and SA. This permits us to better characterize the traits most associated with the realization of an actual suicidal attempt, in order to identify vulnerability to commit a concrete suicide attempt early on.

## Materials and methods

### Design

This clinical cross-sectional study is performed according to the REporting of studies Conducted using Observational Routinely collected health Data (RECORD) (see Supplementary material). This statement received the approval of the Ethics Committee of Policlinico San Matteo in Pavia, Italy (P-20200055757) and was conducted following the Declaration of Helsinki (1964) and its later amendments. The participant's parents gave their written informed consent and were free to withdraw their participation in the study at any time. Moreover, the patients gave their agreement to participate in the study and to the processing of their data. The dataset generated and analyzed during the current study is anonymized and available in the Zenodo repository (14).

### Participants

We recruited 88 Adolescents aged 12–18 years (extremes included) from April 2020 to January 2022. They were admitted to Northern Italian Child Neurology and Psychiatry Service as outpatients or inpatients and presented SI or SA, assessed using the Columbia-Suicide Severity Rating Scale-Children Baseline Screening (C-SSRS). By using this scale, we divided the sample into two groups: patients with a history of SA, who had made an actual or interrupted attempt, and patients without suicidal attempts, presenting SI according to the five questions of the C-SSRS [“Wish to be dead,” “Non-Specific Active Suicidal Thoughts,” “Active Suicidal Ideation with Any Methods (Not Plan) without Intent to Act,” “Active Suicidal Ideation with Some Intent to Act, without Specific Plan,” “Active Suicidal Ideation with Specific Plan and Intent”].

**Figure 1:**
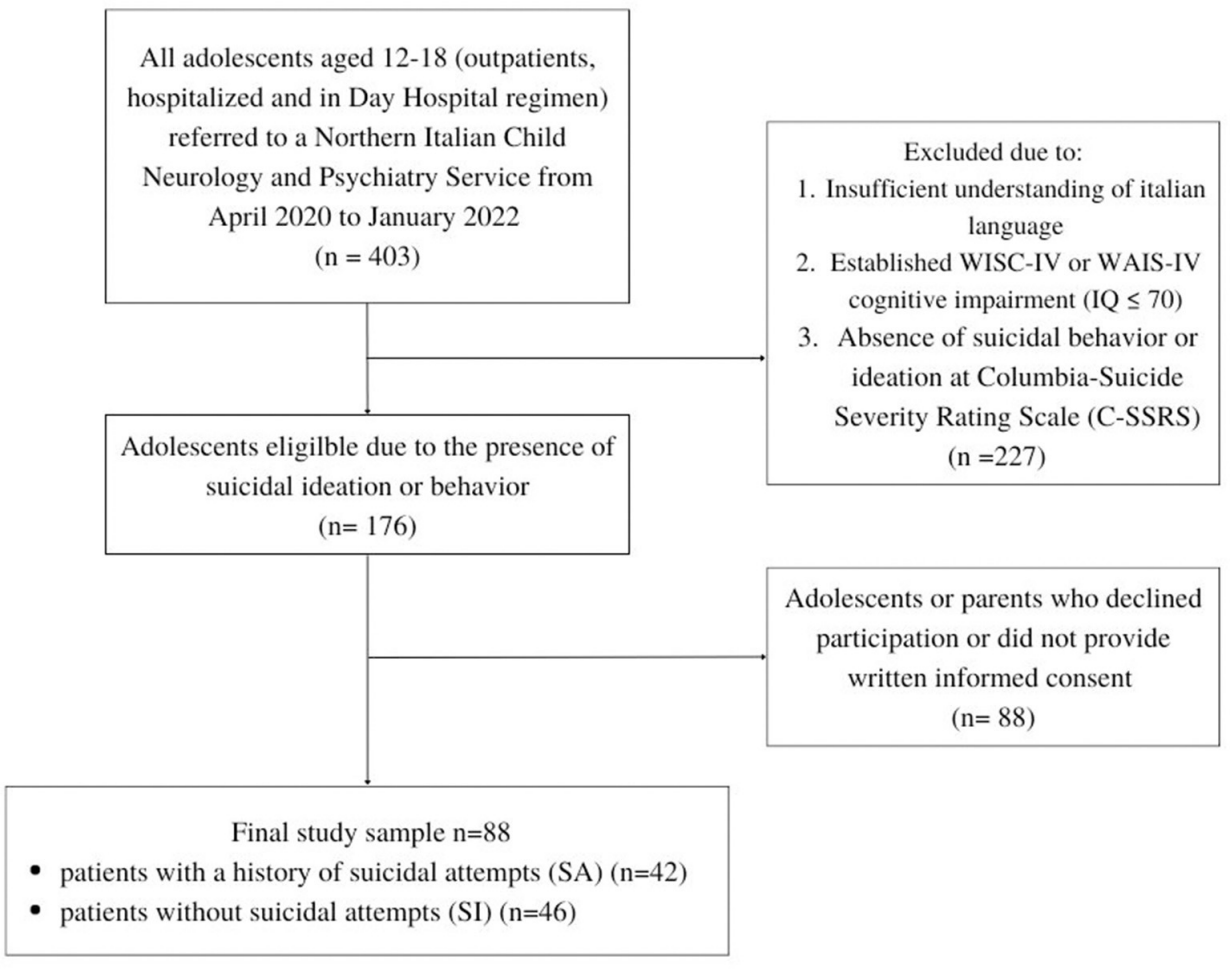
Study sample flowchart.

Patients presenting intellectual disability, or an insufficient understanding of the Italian language were considered not eligible for the study. The absence of intellectual disability was assessed using the appropriate Wechsler intelligence scale according to patients' age (15, 16). Figure 1 shows the study sample flowchart.

### Procedures

All subjects are firstly evaluated by a trained child neuropsychiatrist who collected socio-demographic and anamnestic data.

We assess the presence and severity of SI and/or SA using the Columbia-Suicide Severity Rating Scale-Children Baseline Screening (C-SSRS) (17), a semi-structured interview that determines the presence of both SI and SA lifetime and in the last 6 months, through the following constructs:

- Severity of SI, based on five progressive questions in which 1 = wish to be dead, 2 = non-specific active suicidal thoughts, 3 = active SI with any method (not plan) without intent to act, 4 = active SI with some intent to act, without a specific plan, and 5 = active SI with specific plan and intent. Clinicians investigate the presence of SI lifetime and in the past 6 months (dichotomic response yes/no).
- Intensity of the ideation: explored by considering the frequency of the highest severity degree of ideation reported by the subject (from 1 to 5).
- SA, through a categorical scale that investigates the presence of actual, interrupted, and aborted attempts lifetime (dichotomic response yes/no) and the number of each attempt, and preparatory acts (dichotomic response yes/no).
- Actual and potential lethality of suicidal behavior in case of actual attempts.

Once the presence of SI or SA is determined, a trained neuropsychiatrist or psychologist performs a diagnostic assessment designed to identify the presence of a DSM-5 diagnosis and define the psychological and behavioral characterization of the patients using the following scales:

- DSM-based Kiddie Schedule for Affective Disorders and Schizophrenia (K-SADS-PL DSM-5) (18): a diagnostic interview for the assessment of past and current psychopathological disorders in children and adolescents according to DSM-5 criteria (e.g., Eating disorders, depressive disorders, anxiety disorders OCD, PTSD, etc.);
- Structured Clinical Interview for DSM-5 Personality Disorders (SCID-5 PD) (19): a semi-structured diagnostic interview that evaluates personality disorders according to the groups and DSM-5 diagnostic criteria. Patient fills in a questionnaire with 106 questions indicating whether he/she recognizes himself/herself in the statements or not. Then a trained neuropsychiatrist or psychologist performs an interview to assess the presence of personality disorders.
- Beck Depression Inventory-Short form (BDI) (20): a self-reported questionnaire composed of 13 items that help to identify depressed patients. Each item has a score between 0 and 3, higher scores correspond to greater severity of depressive symptoms (5–7 = mild; 8–15 = moderate; >16 = severe). In the current sample, the reliability coefficient (Cronbach) of BDI is 0.87.
- Children Global Assessment Scale (C-GAS) (21): a 100-point rating scale completed by the clinician. Evaluates the subject's psychosocial and work functioning, placing it on a hypothetical continuum ranging from mental health (91–100 = Superior functioning) to the most serious mental disorder with a risk of death (1–10 = need constant supervision). A functional impairment is usually present with a score of 60 or less.
- Multi-Attitude Suicide Tendency (MAST) (22, 23): a scale used to assess attitude for life and death. This measure, designed to assess suicidal tendencies in youth, is a 30-items scale with score from 1 (Strongly Disagree) to 5 (Strongly Agree). Four subjective attitudes are explored as mediators of suicidality, without distinction between ideation or attempts: attraction to life (AL), repulsion by death (RD), repulsion by life (RL), and attraction to death (AD). In the MAST a score under 3 is considered clinically significant for the AL dimension and a score >3 is considered clinically significant for RL, AD, and RD ones. Osman et al. (24) proved the reliability of the MAST scale to identify the presence of risk factors (high scores on the RL subscale) and protective factors (high scores on the AL subscale) concerning suicidality in adolescence. The RD subscale does not appear to show significant differences between suicidal and non-suicidal subjects and the AD subscale seems to be less reliable because it includes heterogeneous content that may reflect personal beliefs and traditions, in addition to SI. Each scales show acceptable reliability (Reliability coefficients are reported in **Table 3**).

All the clinician-filled scales and semi-structured interviews were carried out but considering the self-report questionnaire we had few missing at random data due to reduced patient cooperativeness or due to depressive states so severe that the patient is unable to complete the questionnaires.

However, previous studies have shown how the combined use of self-report measures can support the clinician in the use of the MAST scale (24, 25). Research conducted by Maggiolini et al. (25) specifically correlates attitudes toward life primarily with internalizing issues. Therefore, we explored this linkage by assessing the presence of risk and protective factors through the MAST scale within patients characterized by internalizing problems at Youth Self-Report (YSR 11-18):

- Youth Self-Report (YSR 11-18) (26) is a self-reported questionnaire intended for 11- to 18-year-olds composed of 112 items. Each item has three possible answers (0 = not true, 1 = sometimes true, 2 = often true). The questionnaire results can be analyzed through different sets of sub-scales to study various aspects of the subject's functioning. The Syndromic scales investigate the presence of Internalizing problems (namely “Anxious/Depressed” “Withdrawn/Depressed” and “Somatic Complaints”), Externalizing problems (“Rule-Breaking Behavior” and “Aggressive Behavior”) and other issues (“Social Problems,” “Thought Problems,” and “Attention Problems”). The whole of these subscales determines an overall index of “Total problems.” Another set of sub-scales to elaborate the questionnaire result is the so-called DSM-Oriented scales which comprehend six categories: “Affective Problems,” “Anxiety Problems,” “Attention Deficit Hyperactivity Problems” Oppositional Defiant Problems,” “Somatic Problems,” and “Conduct Problems.” For Syndromic scales, scores ≤ 64 are considered in the normal range, scores between 65 and 69 (extremes included) are considered borderline scores, and those ≥70 are commonly considered in the clinical range. For those scales indicating Internalizing, Externalizing, and Total Problems, scores ≤ 59 are considered in the normal range, scores between 60 and 63 are considered borderline scores, and those ≥64 are considered in the clinical range.

### Statistical analysis

Descriptive analyses were performed for demographic and clinical characteristics for the total sample and, separately, for the two groups (SI vs. SA). These analyses included mean values and standard deviation (SD), as appropriate for continuous variables, and absolute and relative frequencies for categorical variables.

Descriptive analyses were completed by statistical comparisons between the two groups. Independent *t*-test was used for numerical variables (e.g., age), while the Chi-square test was used for categorical variables (e.g., gender, ethnicity, and SES), complemented by *post-hoc* analyses (Dunn test). Statistical analyses were performed with IBM SPSS version 27.0 (27).

## Results

We enrolled 88 adolescents with SI and/or SA. Table 1 shows sociodemographic and anamnestic data for the total and two subgroups. The two groups were homogeneous in all the categories.

**Table 1:** Sociodemographic and anamnestic data in the total sample and the two subgroups.

	**Total (*N* = 88)**	**SA (*N* = 42)**	**SI (*N* = 46)**	***p^a^***
Female	77 (87.5%)	38 (90.5)	39 (84.8%)	0.420
Mean (SD) age	15.21 (1.63)	15.20 (1.43)	15.22 (1.80)	0.479
**Ethnicity**				0.634
Caucasian	71 (80.7%)	34 (81%)	37 (80.4%)	
Asian	1 (1.1%)	-	1 (2.2%)	
Latin	3 (3.4%)	1 (2.4%)	2 (4.3%)	
African	4 (4.5%)	2 (4.8%)	2 (4.3%)	
Mixed	7 (8%)	3 (7.1%)	4 (8.7%)	
Other	2 (2.3%)	2 (4.8%)	-	
**Family status**				
Separated parents	33 (37.5%)	18 (42.9%)	15 (32.6)	0.278
**Socio-economic status (SES score)**				0.662
Upper Class	12 (13.6%)	7 (16.7%)	5 (10.9%)	
Upper middle	21 (23.9%)	11 (26.2%)	10 (21.7%)	
Lower middle	22 (25%)	10 (23.8%)	12 (26.1%)	
Upper lower	16 (18.2%)	9 (21.4%)	7 (15.2%)	
Lower	10 (11.4%)	3 (7.1%)	7 (15.2%)	
**Family history of psychiatric disorders**				
Psychosis	1 (1.1%)	-	1 (2.2%)	0.623
Depression	10 (11.4%)	7 (16.7%)	3 (6.5%)	0.335
Bipolar disorders	2 (2.3%)	2 (4.8%)	-	0.297
Anxiety disorders	6 (6.8%)	4 (9.5%)	2 (4.3%)	0.557
Substance abuse	-	-	-	0.552
Behavioral disorders	2 (2.3%)	2 (4.8%)	-	0.213
Eating disorders	1 (1.1%)	1 (2.4%)	-	0.156
Obsessive-compulsive disorder	1 (1.1%)	-	1 (2.2%)	0.213
Personality disorders	4 (4.5%)	2 (4.8%)	2 (4.3%)	0.806
Other non-specified psycho-neurological disorders	-	-	-	0.778
**Patient social relationships**				0.597
Proper	27 (30.7%)	12 (28.6%)	15 (32.6%)	
Poor	45 (51.1%)	21 (50%)	24 (52.2%)	
Social withdrawal	15 (17%)	9 (21.4%)	6 (13%)	
**Patient's academic performance**				0.720
Excellent	14 (15.9%)	5 (11.9%)	9 (19.6%)	
Good	34 (38.6%)	16 (38.1%)	18 (39.1%)	
Sufficient	22 (25%)	13 (31%)	9 (19.6%)	
Insufficient	12 (13.6%)	6 (14.3%)	6 (13%)	
School withdrawal	5 (5.7%)	2 (4.8%)	3 (6.5%)	
**Risky behavior**				< 0.001***
Absent	19 (21.6%)	5 (11.9%)	14 (30.4%)	
Nonsuicidal self-injury (NSSI)	42 (47.7%)	12 (28.6%)	30 (65.2%)	
Substance abuse	2 (2.3%)	2 (4.8%)	-	
Attempted suicide	11 (2.5%)	10 (23.8%)	1 (2.2%)	
NSSI+ substance abuse	1 (1.1%)	-	1 (2.2%)	
NSSI+ attempted suicide	11 (12.5%)	11 (26.2%)		
NSSI+ substance abuse + attempted suicide	2 (2.3%)	2 (4.8%)	-	
**Psychopharmacological therapy at admission**				
Antipsychotic drugs	36 (40.9%)	21 (50%)	15 (32.6%)	0.110
Antidepressant drugs	30 (34.1%)	12 (28.6%)	18 (39.1%)	0.262
Benzodiazepines	52 (59.1%)	26 (61.9%)	26 (56.5%)	0.578
Mood stabilizers	17/19.3%)	14 (33.3%)	3 (6.5%)	0.002**
**Psychological therapy at admission**				
Individual psychotherapy	37 (42%)	18 (42.9%)	19 (41.3%)	0.807
Familiar psychotherapy	2 (2.3%)	2 (4.8%)	-	0.130
Parental support	7 (8%)	3 (7.1%)	4 (8.7%)	0.555

^a^*p*: with categorical variables, the *p*-value of the Chi-squared test is reported; with continuous variables, the *p*-value of the t-test is reported. Significance: *p < 0.05; **p < 0.01; ***p < 0.001.

C-SSRS scale has been used to further characterize SI. Patients who had made SA showed higher SI severity, *t*_(86)_ = −3.485, *p* < 0.001 (M_SA_ = 4.10, SD_SA_ = 1.543 vs. M_SI_ = 2.96, SD_SI_ = 1.520). Frequency of ideation was equal among SA and SI groups, *t*_(86)_ = −1.648, *p* = 103 (M_SA_ = 2.86, SD_SA_ = 1.027 vs. M_SI_ = 2.50, SD_SI_ = 1.002) (Table 2).

**Table 2:** Distribution of the C-SSRS intensity of suicidal ideation in the SA and SI groups.

**Group**	**Intensity of ideation**	***N***	**%**
SI (*n* = 46)	0	2	4.3
	1	10	21.7
	2	3	6.5
	3	12	26.1
	4	11	23.9
	5	8	17.4
SA (*n* = 42)	0	2	4.8
	1	3	7.1
	2	2	4.8
	3	3	7.1
	4	4	9.5
	5	28	66.7

The psychological characteristics of the total sample and the two groups performed using the SCID-5 PD and K-SADS-PL DSM-5 interviews are reported in Supplementary Table S1.

The two groups did not differ in terms of diagnoses at admission based on the K-SADS-PL DSM-5 Interview. On the other hand, in the SA group, the proportion of personality disorders was higher than in the SI group [$\chi_{\left(3\right)}^{2}$ = 8.775, *p* = 0.032].

Exploring subject's psychosocial and work functioning through the C-GAS scale, no significant between-group differences emerged (M_SA_ = 47.08, SD_SA_ = 12.45; M_SI_ = 48.09, SD_SI_ = 9.68).

Significant differences emerge instead when investigating levels of subjective depression within the two groups using the BDI questionnaire: in our sample patients of the SA group experienced higher depression levels [M_SA_ = 24.89, SD_SA_ = 7.64; M_SI_ = 19.86, SD_SI_ = 9.68; *t*_(70)_ = −2.65, *p* = 0.01].

The use of YSR 11-18 does not seem to show any difference in psychological and behavioral characterization between the two groups in any of the subscales. We notice an overall prevalence of internalizing problems compared to externalizing ones (Table 3).

**Table 3:** Psychological characterization of the two groups using YSR 11-18 questionnaire.

	**SI**		**SA**		**Cronbach's α**
	**M**	**SD**	**M**	**SD**	
Total problems	**67.62**	9.56	**69.35**	10.86	0.78
Internalizing problems	**74.13**	8.93	**74.83**	11.05	0.86
Externalizing problems	55.82	9.23	57.83	10.83	0.86
Anxious/Depressed	**74.44**	11.16	**77.78**	10.97	0.81
Withdrawn/Depressed	**69.36**	10	**71.68**	12.61	0.77
Somatic complaints	**65.16**	9.42	64.35	8.99	0.67
Social problems	64.71	10.39	**67.08**	9.64	0.72
Thought problems	**68.96**	10.41	**71.45**	9.95	0.70
Attention problems	62.6	9.505	62.98	10.18	0.68
Rule-breaking behavior	56.87	8.05	59.28	9.09	0.78
Aggressive behavior	57.24	7.44	58.18	7.82	0.76
Affective problems	**76.11**	10.61	**78.72**	10.82	0.74
Anxiety problems	63.93	8.91	63.31	9.03	0.66
Somatic problems	**67.53**	8.53	**67.97**	7.51	0.62
Attention deficit/Hyperactivity problems	58.18	6.26	58.79	6.35	0.61
Oppositional defiant problems	57.84	7.50	57.31	7.82	0.54
Conduct problems	56.09	7.81	59	9.18	0.73

Bold clinical and borderline scores. Cut-offs are reported in the Procedures section.

Although the use of the MAST scale alone does not allow us to draw differences into the sample groups as well (Table 4), as previous literature stated (28), the study of cross-correlation between MAST and YSR highlights significant results. Patients who present Internalizing Problems at the YSR show a prevalence of Repulsion by Life (RL) in both SA and SI groups. Regarding Attraction to Life (AL), AL is negatively related to internalizing profiles at YSR only for patients with SA (Table 5).

**Table 4:** Characterization of suicidal attitude using the MAST scale in the two groups.

	**SI**		**SA**		**Cronbach's α**
	**M**	**SD**	**M**	**SD**	
AL	2.47	0.62	2.67	0.74	0.80
RL	3.57	0.67	3.57	0.61	0.69
AD	3.48	0.70	3.30	0.60	0.77
RD	2.11	1.01	1.98	0.81	0.92

AL, attraction to life; RL, repulsion by life; AD, attraction to death; RD, repulsion by death.

AL significance cut off < 3, RL, AD, RD significance cut off >3.

**Table 5:** Characterization of suicidal attitude using MAST scale in patients presenting internalizing problems (INT) at YSR 11–18.

**Group**		**MAST AL**	**MAST RL**	**MAST AD**	**MAST RD**
SI YSR INT (*n* = 42)	Pearson correlation	−0.27	0.41**	0.10	−0.12
SA YSR IN (*n* = 39)	Pearson correlation	−0.71**	0.67**	0.27	−0.19

The ** symbol indicate the Pearson Correlation (r).

## Discussion

In this work, we studied a sample of patients presenting SI to better characterize the features most frequently associated with SA.

As expected, patients with SA, compared to those with SI, showed higher SI severity according to the C-SSRS. Hence a greater intensity and structuring of the ideation with the appearance of intent and planning seems to be positively associated with the likelihood of an actual suicidal attempt (29–31). On the contrary, the frequency of ideation does not seem to differentiate the two groups. These findings support the role of the plan, not the frequency of SI, as a possible marker of increased risk of SA. They also stress the importance of C-SSRS to assess the severity of SI in comparison with other scales (32) for its clinical implications.

The objective observation of our sample carried out by the clinician using semi-structured interviews (K-SADS-PL DSM-5 and SCID-5 PD) did not show substantial differences between the two groups except for the prevalence of personality disorders in patients of the SA group. A deeper distinction between different personality disorders couldn't be explored due to the limited sample size and may be the object of further studies.

On the other hand, patients belonging to the SA group reported more intense depressive symptoms in the self-report questionnaires than in the other group. The discrepancy between clinical observation and what is subjectively reported by the patients suggests that particular attention should be paid to their subjective experience. They may indeed find it more difficult to express their suffering in front of the clinician than in filling out a questionnaire (33, 34).

In our sample, most patients reported Internalizing Problems at YSR, without distinctions between SI and SA. According to the study conducted by Masi et al. (28), these groups did not even differ concerning their attitude toward life and death, related their fearlessness about death and to the capability for suicide, investigated using the MAST scale.

Exploring the presence of possible protective and risk factors on the MAST scale in the group of patients who reported internalizing problems at YSR, our data are in line with those observed by Maggiolini et al. (25), namely a positive association between SI and an attitude of RL. RL is also positively associated with YSR Internalizing Problems for the SA group. These findings are rather intuitive, considering that Repulsion to Life is commonly considered a risk factor for committing a suicide attempt.

The finding that AL is negatively associated with YSR Internalizing problems only for SA supports the possibility to explore this prevalent attitude for life. Considering that in literature results regarding internalizing profiles in adolescence are not unanimous (28, 35), we hypothesized that the presence of internalizing profiles in adolescence associated with a low score in AL deserves further investigation because it may turn out to represent a possible vulnerability factor helpful in predicting the transition from SI to SA.

It is necessary to remark that since our research does not include any subject dead by suicide, the analysis of nonfatal suicidal acts might be limited in identifying the elements that lead to lethality. Indeed, while only a minority of attempers die by suicide, a lethal outcome happens more often during the first SA (12, 36, 37).

Another important limitation is represented by the fact that the characterization of the SA group was carried out *a posteriori* concerning the suicidal act. Thus, we cannot state with certainty that what emerged in our sample reflects a pre-existing risk condition.

These are both major limitations in suicide research because people who commit SA can only be recruited and analyzed retrospectively. Carrying on a characterization of subjects focusing on days and hours preceding the act may allow overcoming this limitation (38, 39).

A final limitation of our study is that we recruited our sample in a period that overlaps with the COVID-19 pandemic health emergency, so it is possible that the characteristics we identified were more specifically associated with this historical period and therefore less general.

In our sample, we did not use instruments to assess impulsivity. Several studies (40–42) underline the importance to differentiate between aggression and self-aggression in order to explore the relationship with impulsivity in order to identify individuals who are more likely to attempt suicide impulsively. We only have the data provided by the YSR regarding aggression, which attests that the mean scores in both SA and SI are subthreshold (nonclinical value). This aspect, therefore, needs further investigation. In line with more recent theories, our analysis emphasizes the importance of pursuing a careful distinction between SI and SA in suicide research.

Finally, recent literature (43) has stimulated us to think that it would be interesting to replicate our findings in a more homogeneous sample to explore gender differences as well.

## Conclusions

Our results indicate that both adolescents with SI and adolescents with SA have high scores on specific measures. They also suggest the importance of studying the nature of the relationship between the instruments used in the assessment to identify possible vulnerability factors in adolescence useful in predicting the transition from SI to SA.

Regarding clinical practice, our findings have a strong impact. They suggest the importance of using self-report and clinician-administered tools (e.g., C-SSRS and MAST). Not only are these instruments easy and quick to administer, but they could also provide consistent support for targeting, from the earliest assessment, adolescents at higher risk of enacting SA, thus enabling further investigations and, where necessary, early intervention. Considering the above, our results improve the foreseeabilty of SA by expanding current knowledge about the formulation of suicide risk (44). Concluding, this study offers an effective methodology for assessing at-risk adolescents presenting SI and SA.

## Data availability statement

The datasets presented in this study can be found in online repositories. The names of the repository/repositories and accession number(s) can be found below: https://doi.org/10.5281/zenodo.6516856, Zenodo repository.

## Ethics statement

The studies involving human participants were reviewed and approved by Ethics Committee of Policlinico San Matteo in Pavia, Italy (P-20200055757). Written informed consent to participate in this study was provided by the participants' legal guardian/next of kin.

## Northern Italian suicidality research group

Francesca Comi, Francesca Esposito, Grazia Giana, Elisa Lazzaroni, Irene Orlandi, Flavia Passoni, Antonio Piotti, Chiara Rogantini, Francesca Rossicone, Arianna Vecchio, and Marina Zabarella.

## Author contributions

MM, RI, and LC designed the study. CC, MO, RI, PG, and IR collected data. EC conducted statistical analyses. RB and OM provided scientific supervision. CC, RI, and MO drafted the manuscript. All authors revised the final version and agreed on the paper to be submitted for publication.

## Funding

The present study was supported by the Italian Ministry of Health (Ricerca Corrente).

## Conflict of interest

The authors declare that the research was conducted in the absence of any commercial or financial relationships that could be construed as a potential conflict of interest.

## Publisher's note

All claims expressed in this article are solely those of the authors and do not necessarily represent those of their affiliated organizations, or those of the publisher, the editors and the reviewers. Any product that may be evaluated in this article, or claim that may be made by its manufacturer, is not guaranteed or endorsed by the publisher.
