# Supplementary material for: Psychological and behavioral characterization of suicide ideators and suicide attempters in adolescence
Source: Front Psychiatry. 2022 Oct 10;13:1009460. doi: 10.3389/fpsyt.2022.1009460 (PMC9589341; doi:10.3389/fpsyt.2022.1009460)
Supplement: Supplementary file 1 [file Table_1.DOCX]

**Table S1.** Psychological characterization of the total sample and the two groups by SCID-5 PD and K-SADS interviews.

|  | **Total (N=88)** | **SA (N=42)** | **SI (N=46)** | ***p*** |
| --- | --- | --- | --- | --- |
| Diagnosis of Personality Disorder (PD) - SCID-5 PD results |  |  |  | .006 |
| *PD non detected* | 11(12.5%) | 1(2.4%) | 10(21.7%) |  |
| *PD detected* | 77(87.5%) | 41(97.6%) | 36(78.3%) |  |
| Diagnosis of psychopathological disorders - K-SADS results | | | | |
| *Depressive disorders* | 67(76.1%) | 30(71.4%) | 37(80.4%) | .062 |
| *Manic episode* | 5(5.7%) | 2(4.8%) | 3(6.5%) | .160 |
| *Hypomanic episode* | 2(2.3%) | 1(2.4%) | 1(2.2%) | .881 |
| *Disruptive mood dysregulation disorder* | 1(1.1%) | 1(2.4%) | - | .488 |
| *Psychotic disorder* | 9(10.2%) | 5(11.9%) | 4(8.7%) | .878 |
| *Panic disorder* | 14(15.9%) | 10(23.8%) | 4(8.7%) | .054 |
| *Agoraphobia* | 2(2.3%) | 1(2.4%) | 1(2.2%) | .963 |
| *Separation anxiety disorder* | 5(5.7%) | 3(7.1%) | 2(4.3%) | .762 |
| *Social Anxiety* | 16(18.2%) | 9(21.4%) | 7(15.2%) | .507 |
| *Specific phobia* | 5(5.7%) | 2(4.8%) | 3(6.5%) | .512 |
| *Generalized anxiety disorder* | 19(21.6%) | 9(21.4%) | 10(21.7%) | .624 |
| *Obsessive-compulsive disorder* | 7(8%) | 3(7.1%) | 4(8.7%) | .863 |
| *Eating disorder - anorexia* | 21(23.9%) | 9(21.4%) | 12(26.1%) | .566 |
| *Eating disorder – binge eating* | 2(2.3%) | - | 2(4.3%) | .231 |
| *Eating disorder – bulimia nervosa* | 2(2.3%) | 1(2.4%) | 1(2.2%) | .797 |
| *ADHD* | - | - | - | .092 |
| *Oppositional defiant disorder* | 1(1.1%) | - | 1(2.2%) | .067 |
| *Conduct disorder* | 1(1.1%) | - | 1(2.2%) | .346 |
| *Tic disorder* | - | - | - | .293 |
| *Autism spectrum disorder* | - | - | - | - |
| *Tobacco abuse* | 2(2.3%) | 1(2.4%) | 1(2.2%) | .393 |
| *Alcohol abuse* | - | - | - | .612 |
| *Substances abuse* | 2(2.3%) | 2(4.8%) | - | .312 |
| *PTSD* | 1(1.1%) | 1(2.4%) | - | .423 |

Significance: **p*<.05; ** *p*<.01; ****p*<.001
